# Gene and Base Editing as a Therapeutic Option for Cystic Fibrosis—Learning from Other Diseases

**DOI:** 10.3390/genes10050387

**Published:** 2019-05-21

**Authors:** Karen Mention, Lúcia Santos, Patrick T. Harrison

**Affiliations:** 1 Department of Physiology, University College Cork, Cork T12 K8AF, Ireland; karen.mention@gmail.com (K.M.); lasantos@fc.ul.pt (L.S.); 2 University of Lisboa Faculty of Sciences, BioISI—Biosystems & Integrative Sciences Institute, 1749-016 Lisboa, Portugal

**Keywords:** gene editing, base editing, cystic fibrosis, AAV, ribonucleotide particle, guideRNA, CRISPR, Cas9

## Abstract

Cystic fibrosis (CF) is a monogenic autosomal recessive disorder caused by mutations in the *CFTR* gene. There are at least 346 disease-causing variants in the *CFTR* gene, but effective small-molecule therapies exist for only ~10% of them. One option to treat all mutations is CFTR cDNA-based therapy, but clinical trials to date have only been able to stabilise rather than improve lung function disease in patients. While cDNA-based therapy is already a clinical reality for a number of diseases, some animal studies have clearly established that precision genome editing can be significantly more effective than cDNA addition. These observations have led to a number of gene-editing clinical trials for a small number of such genetic disorders. To date, gene-editing strategies to correct *CFTR* mutations have been conducted exclusively in cell models, with no in vivo gene-editing studies yet described. Here, we highlight some of the key breakthroughs in in vivo and ex vivo gene and base editing in animal models for other diseases and discuss what might be learned from these studies in the development of editing strategies that may be applied to cystic fibrosis as a potential therapeutic approach. There are many hurdles that need to be overcome, including the in vivo delivery of editing machinery or successful engraftment of ex vivo-edited cells, as well as minimising potential off-target effects. However, a successful proof-of-concept study for gene or base editing in one or more of the available CF animal models could pave the way towards a long-term therapeutic strategy for this disease.

## 1. Introduction

Cystic fibrosis (CF) is a monogenic autosomal recessive disorder caused by mutations in the Cystic Fibrosis Transmembrane Conductance Regulator (*CFTR*) gene which encodes a chloride/bicarbonate channel expressed on the apical surface of secretory cells. Lack of CFTR activity results in multiorgan dysfunction and ultimately mortality from respiratory sequelae. For more than seven decades, therapies for CF focused on treatment of the symptoms rather than the origin of the disease which is the CFTR protein dysfunction [1]. However, since 2011, a number of small-molecule therapies known as potentiators and correctors have been developed and licenced that can functionally correct at least 33 different CF-causing mutations [2], accounting for 90% of individuals with CF. However, based on the March 2019 data from cftr2.org [3], this still means 90% of disease-causing variants cannot be corrected by any available small-molecule therapies.

An alternative therapeutic approach that has been extensively studied over the last 25 years is the use of the CFTR cDNA, delivered directly to the lung, to complement the genetic defect in *CFTR*. This approach could potentially be curative, at least for CF lung disease, in a manner that is independent of the disease-causing variants. However, after more than 20 clinical trials, the only clinical benefit reported so far has been a stabilisation of lung function in patients who received at least nine doses of CFTR cDNA delivered by cationic liposomes over a 12-month period [4]. In contrast, both in vivo and ex vivo gene therapy approaches have been very successful in a wide range of other diseases over the same time frame [5]. It is not clear why CFTR cDNA-based therapy has not yet been successful, but a number of studies discussed in detail below have shown that precision editing of the genome can be more effective than cDNA addition, at least in animal models [6,7].

So, could the precision repair of CF-causing variants be a feasible alternative to cDNA addition? Perhaps the first question to address is how such changes can be made in the genome in the first instance. The magnitude of the challenge was succinctly captured by Joshua Lederberg in his 1959 Nobel Prize Lecture, where he stated “The *ignis fatuus* of genetics has been the specific mutagen, the reagent that would penetrate to a given gene, recognize and modify it in a specific way” [8]. Having acknowledged that it may be possible to exploit base-pairing to potentially find a target site in a genome, and that it would need to be highly specific to avoid off-target effects, he also noted that ‘no chemical reagent capable of substituting one nuclein for another in the structure of existent DNA’ had yet been recognised.

Over the next sixty years, many reagents were developed, based partly on Lederberg’s discoveries concerning genetic recombination, to create targeted, precise and permanent changes to the cellular genome, culminating in the ability to generate mice from ES cells engineered with precise modifications in their genome [9]. However, the efficiency of this approach was considered too low for therapeutic application. It took a key proof-of-concept experiment from Maria Jasin that a targeted double-stranded break (DSB) could substantially increase editing efficiency [10], and the development of a new set of synthetic reagents called zinc finger nucleases (ZFNs) [11] to lay the foundations for therapeutic editing. But it would take another decade before fully programmable ZFNs were used to correct a disease-causing mutation in human cells [12]. The ZFNs catalyse the initial step in gene repair, the formation of the DSB, which can then be repaired using sequence information from an exogenous donor molecule to introduce the desired change in the genome mediated by the cellular homology-directed repair (HDR) pathway. Subsequently, the TAL effector nucleases (TALENs) and the Clustered Regularly Interspaced Short Palindromic Repeats (CRISPR) encoded, RNA-guided Cas family of nucleases have provided a diverse number of “specific mutagens” for efficient DNA repair, or, as we now know it, precision gene editing (reviewed in [13,14,15].

While gene editing is already being evaluated in a number of FDA-approved clinical trials, there are still many challenges to be addressed. First, DSBs created by ZFNs, TALENs and CRISPR nucleases are not always repaired via the HDR pathway which is necessary for precision editing, rather they can also be repaired by a second pathway known as non-homologous end-joining (NHEJ) which can result in unwanted insertions or deletions (referred to as indels) which can reduce the overall effectiveness of the editing process. Second, each of the systems can cause unwanted DSBs at other sites in the genome which share substantial homology to the target site. The repair of these DSBs can give rise to potentially disruptive indels at these sites, or in some cases trigger genomic rearrangements [16]. Several other challenges exist in the therapeutic development, not least the delivery of gene-editing reagents to the correct target cells in vivo, the ability to efficiently engraft cells which are edited ex vivo, and the possibility of immune responses to editing tools in vivo [17,18] which may restrict options for repeated rounds of editing. Finally, given that most monogenetic diseases are usually caused by a wide range of variants within a single locus, there has been substantial focus on strategies which can correct a large number of variants with a single set of reagents. Such strategies may be required for clinical application.

In this review, we highlight some of the key breakthroughs that have been made during the development of gene editing for other diseases, and discuss how they might be applied to cystic fibrosis from a therapeutic perspective. The impact of gene editing on the study of cystic fibrosis [19], other gene-based approaches to treating CF [20,21] and the ethics of somatic cell editing [22] are discussed elsewhere.

## 2. In Vivo Editing—From Mouse to Man in Six Years

In this section, we introduce the first in vivo demonstration that gene editing could correct a disease-causing mutation in a mouse model of haemophilia B in 2011 and discuss how this laid the foundation for a number of therapeutic gene-editing trials in humans just six years later. We also consider how lessons from these studies might be applied to the development of gene editing as a therapeutic approach for CF.

Haemophilia B is a chronic liver disease caused by mutations in the *F9* gene, leading to a severe depletion of coagulation factor IX protein. Consequently, blood clotting cannot be properly regulated in response to injury, resulting in continuous bleeding that can be difficult to control. The disease can be managed by regular intravenous injections of recombinant factor IX, so long as circulating levels of protein are maintained at ~5% of the normal value. As with the majority of genetic disorders, haemophilia B can be caused by any one of a large number of different variants within the target gene. While early gene-editing studies with zinc finger nucleases (ZFNs) showed that it was feasible to correct essentially any single disease-causing variant by the donor-dependent HDR pathway for a number of cell models of genetic disease [23,24] including the F508del variant in *CFTR* [25], this approach does not allow for the complete coverage of the wide spectrum of disease-causing variants.

**Figure 1:**
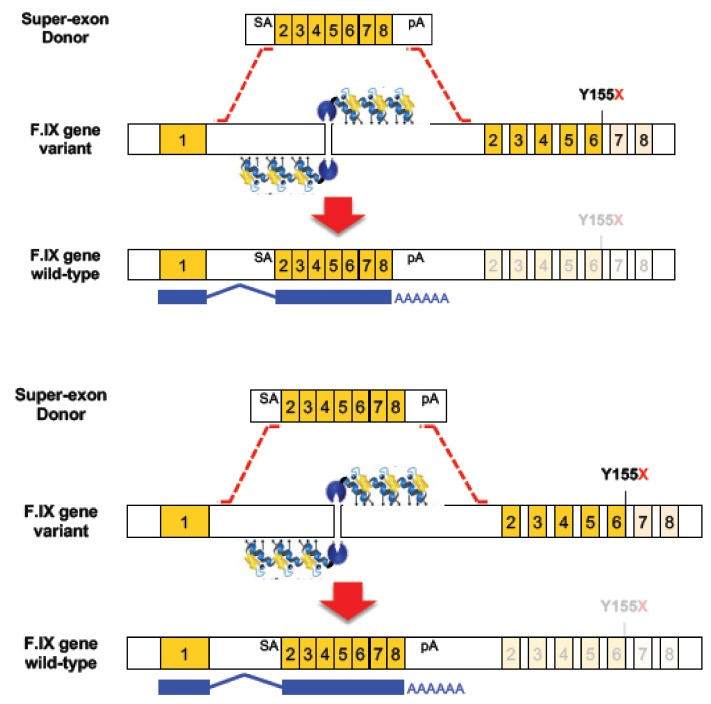
Schematic overview of zinc finger nuclease (ZFN)-mediated homology-directed repair (HDR). Upper panel: superexon donor with homology arms, SA, exons 2–8 fused as partial cDNA and poly A. Middle panel: ZFNs create DSB in intron 1 which triggers strand invasion of donor leading to HDR. Lower panel: superexon incorporated into the genome such that mRNA splices from endogenous exon 1 to superexon 2–8. The expression of the corrected mRNA is driven by the endogenous promoter. Adapted from [6].

Faced with the challenge of developing multiple ZFN/donor combinations for each disease-causing mutation in the human *F9* gene, Kathy High and colleagues [6] used ZFN-HDR with a novel form of donor widely known as a superexon, or partial cDNA. As shown in Figure 1, they created a donor comprising exons 2 to 8 (where 95% of the Hb-causing mutations are found), flanked by a splice acceptor and a polyA site, and containing homology arms of ~800 bp either side of the construct. They designed a pair of high-specificity ZFNs to introduce a targeted DSB in the first intron of the *F9* gene to drive the precise and stable integration of the SA-superexon-polyA donor into the genome, which would in turn yield the production of a full-length and functional mRNA. In principle, this approach could correct any mutation localised downstream of exon 1.

When evaluated in human hepatocyte cell lines, they observed evidence of ZFN DSB formation as measured by indels in 45% of alleles, and superexon integration in 17% of alleles. For assessment in vivo, a humanized mouse model of haemophilia B was used containing the *hF9* gene with a premature stop codon in exon 6 (Y155X). Mice received intraperitoneal injections with two different AAV8 vectors, one to deliver the ZFN pair and the other one to deliver the superexon donor. This resulted in a targeting efficiency of ~2% in liver which was stable for at least one month post-injection, and circulating levels of hF.IX of 2 to 3% of wild-type level which was sufficient to restore normal haemostasis. Two types of off-target effects were observed in this study. First, the ZFN pair created a low level of indels at one of the 20 potential off-target sites. Second, some random adeno-associated virus (AAV) integration events were observed in the mouse genome though not at, or adjacent to, known oncogenic sites, and some direct AAV vector integration was found in the cut site [6].

Building on observations from this study, Barzel and colleagues [26] developed a novel strategy to determine if a hF9 superexon could be expressed from an alternate genomic locus as a step towards a universal platform for expression of any secreted protein. They chose the *Alb* locus in part due to its strong liver-specific promoter, postulating that even a low level of integration could enable high levels of superexon encoded protein production. They designed their targeting construct to avoid the disruption of albumin production by fusing a 2A peptide linker to the *F9* cDNA, integrating the construct just upstream of the *albumin* stop codon [26]. With safety as a major concern for the development of a platform suitable for clinical use, they chose an integration strategy that was driven solely by the homology arms and the AAV vector; no ZFNs or other nucleases were used. The absence of site-specific nucleases may account for the relatively low level of integration observed; only 0.5% of the albumin alleles in hepatocytes were targeted when the AAV9-*hF9* vector was administered to neonatal and adult mice. However, even with this low level of site-specific integration, the hF.IX plasma level was found to be 7 to 20% of normal and the expression was both stable, and restored normal coagulation [26].

As a further step towards clinical development, High and colleagues demonstrated the versatility of the *Alb* locus as a safe harbour for superexon transgene expression. They showed that any one of a number of different human cDNA sequences encoding essential secretory enzymes could be integrated using the same ZFN pair to create a targeted DSB in intron 1 of the *Alb* locus to promote integration; an added advantage was that the desired transgene-targeting constructs could integrate by either HDR or NHEJ [27]. The ability to integrate by both mechanisms, is an important design feature as it enables integration to occur not only in dividing cells (where HDR is active during the S/G2 phase of the cell cycle), but also in non-dividing cells where only the NHEJ pathway is active. Another feature of the design is that the “fusion transcript” created by the splicing of exon 1 from the endogenous *Alb* locus to the integrated superexon means that the proteins were synthesised with the albumin signal peptide. This signal peptide directs the secretion of the proteins from the liver cells, but it is subsequently removed, therefore limiting its impact on the protein of interest’s function. Other strengths of this approach are the fact that the same ZFN pair could be used to stably integrate the superexon of essentially any secretory protein. Subsequently, proof-of-concept studies have established that this platform can correct several lysosomal storage diseases including Mucopolysaccharidosis Type II (Hunter syndrome) [28] and MPS I (Hurler syndrome) [29]. These experiments culminated in the approval of two human in vivo gene-editing trials for MPS I (NCT02702115) and MPSII (NCT03041324), with Brian Madeux becoming the first person to receive gene-editing agents in November 2017, just six years after the first published in vivo data from a mouse model.

### Towards In Vivo Editing with CFTR Superexons to Correct Multiple CF-Causing Variants

The superexon technique could be very useful to correct CF-causing variants, though not necessarily from the *Alb* locus. For example, 92% of all CF-causing alleles (representing 64% of known CF-causing variants) could be corrected with a superexon donor comprising exons 11 to 27, a suitable splicing acceptor site and flanked by homology arms. Such a construct would be small enough to be delivered by AAV vectors, similar to those used in the studies shown above. Proof-of-concept that ZFNs and such a CFTR superexon could correct the F508del variant was reported by Toni Cathomen and colleagues in a bronchial epithelial cell line [30]. Although the efficiency was low, the isolation of edited cells confirmed that the superexon had been precisely integrated into the *CFTR* locus by HDR and could functionally restore CFTR ion channel activity.

Several options to substantially increase the efficiency of superexon integration have been described using NHEJ-based integration strategies. For example, the CRISPR-based homology-independent targeted integration (HITI) strategy which preferentially integrates the superexon in the correct orientation was used to successfully restore a large deletion in the Mertk gene in a rat model of Retinitis pigementosa [7]; we have outlined a HITI-based strategy to correct CF variants with a superexon 11–27 donor [22]. Other potential CRISPR-based methods which may prove suitable are “knock-in blunt ligation” (KiBL) [31] and targeted integration with linearised dsDNA (TILD) [32].

Another challenge for CF is that a superexon 2–27 donor, to correct virtually all known CF-causing variants, would be slightly too large for delivery by standard AAV vectors. However, other non-viral and viral delivery approaches may be suitable (see [22]), including large capacity helper-dependent adenovirus vectors (HD-Ad) which have been successfully used to deliver the CFTR cDNA to lung basal cells [33]. Indeed, these HD-Ad vectors have recently been used to drive the stable integration of CFTR cDNA into the AAVS1 safe harbour site using TALENs [34] or Cas9/gRNA [35]. Moreover, the editing events which drive integration result in the concurrent elimination of TALEN/Cas9 expression, thus potentially reducing off-target effects and risk of immune response to editing machinery.

## 3. Ex Vivo Approaches for Gene Editing in Animal Models

An alternative to in vivo gene editing is to remove target cells from a patient, edit them ex vivo in a controlled laboratory setting and then reimplant them. This autologous ex vivo cDNA addition approach is already clinically well established for a small number of genetic diseases of the immune system [36]. The use of ex vivo gene editing offers a number of potential advantages over in vivo editing such as the potential for selection to enrich edited populations of cells, and the assessment of off-target effects prior to implantation. There are several challenges in developing an autologous gene-edited cell-based therapy for CF, but a recent review article suggests delivery and engraftment may ultimately be feasible [37]. So, here, we focus on some of the editing strategies which are being explored for other diseases where the delivery and engraftment route is well understood, and ask what lessons can be learned in order to develop editing for CF.

X-linked chronic granulomatous disease results in a severe malfunction of the immune system and is caused by mutations in a number of different genes which encode subunits of the NADPH oxidase complex. If NADPH oxidase cannot assemble or function properly, phagocytes are unable to kill foreign invaders and neutrophil activity is dysregulated, leaving affected individuals vulnerable to many types of infection and excessive inflammation. The disease can be treated by autologous gene-modified cells in combination with nonmyeloablative conditioning. However, at least three of 13 patients who have undergone this procedure developed myelodysplastic syndrome due to insertional activation of MDS1–EVI1 [38].

This prompted Malech and colleagues [39] to develop a strategy for ZFN-targeted integration of the gp91phox cDNA into the AAVS1 safe harbour locus in human CD34+ hematopoietic stem and progenitor cells (HSPCs). Optimal conditions for editing occurred when HSPCs were electroporated with mRNAs encoding the ZFNs, then immediately transduced with an AAV6 vector containing the gp91phox expression construct under the control of the MND promoter (which drives high levels of transgene expression in HSPCs). Targeted integration was observed in 7% of alleles, restoring the overall level of p91phox activity to 15% of that observed in a population of CD34+ HSPCs from healthy donors. Of note, indels were detected in a further 21% of AAVS1 alleles but are not predicted to have a deleterious effect. To determine the effects in vivo, an unenriched population of ZFN/donor-treated cells were transplanted into immune-deficient NOD-scid IL2Rγ^null^ (NSG) mice. At eight weeks post-transplant, high levels of engraftment were detected with >10% of the CD45+ cells derived from the ZFN/donor-treated population expressing gp91 [39]. Given that all the genome-editing reagents in the study can be produced to GMP standards, this approach could be suitable for clinical development.

### Towards Ex Vivo Editing with CFTR

While delivery and engraftment may ultimately be feasible [22,37], what are the ex vivo editing options for CF? An early study established the feasibility of integrating the whole CFTR cDNA into the CCR5 safe harbour locus using ZFNs in human iPS cells, resulting in stable expression of mature CFTR band C protein [40]. In addition, several groups have successfully used ZFNs, TALENs and CRISPR to correct individual CF-causing mutations in human iPS cells and confirmed that this restores CFTR activity [41,42,43,44]. However, in all cases, efficiency was relatively low and even though selection, enrichment and differentiation strategies were successfully developed, these strategies may be challenging to scale up for clinical development.

Recently, Porteus and colleagues [45] have taken a different approach, avoiding iPSc and enrichment strategies, and focusing directly on developing the high efficiency editing of a single CF-causing mutation in primary upper airway basal stem cells (UABCs). HDR editing was performed by electroporation of Cas9 and gRNA as a ribonucleoprotein (RNP) complex, immediately followed by transduction with an AAV6 vector carrying the correction template. Under optimal conditions, precision editing was observed in 43% of alleles [45]. This high efficiency editing also resulted in a high level of restoration of CFTR functional activity. While their feeder-free, selection-free system is compatible with a clinical approach for autologous cell-based therapies for CF, it should be noted that the optimal level of precision repair by HDR (43%) was accompanied by an almost equally high level of on-target NHEJ events (38%). While a number of FDA-approved drugs have been shown to improve the HDR:NHEJ ratio [46], it may be time to consider an alternative gene-editing strategy which can yield very high levels of the on-target editing of individual mutations, but with NHEJ levels at least 10-fold lower than conventional HDR. This new technique, first described in the genome of mammalian cells in 2016, is known as base editing.

## 4. DNA Base Editing

**Figure 2:**
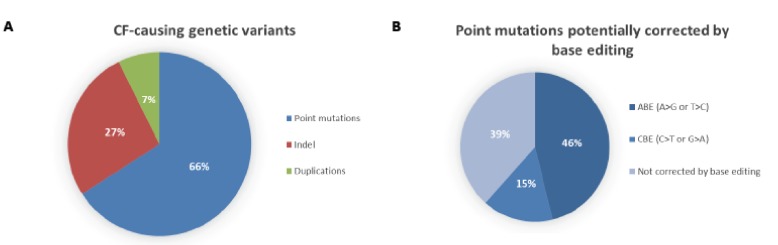
Distribution of cystic fibrosis-causing variants. (**A**) According to the Variant List History from 11 March 2019 available on the CFTR2 database (https://www.cftr2.org/), there are 346 CF-causing variants distributed into three main categories: point mutations (66%), insertions/deletions (Indel, 27%) and duplications (7%). (**B**) Of all the point mutations listed on the CFTR2 database, 46% can potentially be corrected using Adenine Base Editing (ABE) and 15% using cytosine Base Editing (CBE). This prediction only takes into account the nucleotide change that causes the mutations and it does not necessarily mean a suitable PAM sequence is located at the correct spacing from the editing window.

**Figure 3:**
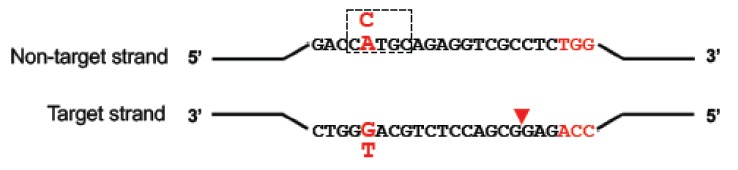
Schematic of base editing. Depending on the base editor used (CBE or ABE), cytosine (C, in blue) or adenine (A, in blue) in a ~5-nt editing window (dashed box) on the non-target strand can be deaminated and changed to a uracil or inosine (not represented), respectively. Nicking of the target strand by nCas9 in the base editor (red triangle) promotes DNA repair and incites the cell to use edited strand as a template. The uracil is then read as a thymine and the inosine as a guanine, converting a C-to-T or a A-to-G.

DNA base editing is a novel genome-editing technique that allows the direct conversion of cytosine to thymine, or adenine to guanine in genomic DNA, in a programmable manner, without generating DSBs. The process requires no donor DNA template and, as it does not rely on HDR, it can occur at all stages of the cell cycle, making it suitable for use in non-dividing cells. More than 50% of known CF-causing SNPs in *CFTR* (see Figure 2), which equates to ~12% of alleles in the CFTR2 database [3], are caused by T>C, G>A, C>T or A>G changes. If the T>C or G>A variants on the coding strand are located in a suitable editing window relative to a functional protospacer adjacent motif (PAM) sequence (see Figure 3), then they could be corrected by base editing with very low levels of unwanted indels. For the C>T and A>G variants, the editing would have to be initiated on the opposite strand, again with a requirement that the target base is located in a suitable editing window relative to a functional PAM sequence. Here, we describe the two major classes of base editors and selected experiments where base editing has been used to establish therapeutic proof-of-principle for this approach in mouse models of a number of genetic diseases.

Currently, there are two major types of base editors, those that convert C to T and those that convert A to G. The DNA base editors typically consist of a fusion protein which comprises a catalytically impaired CRISPR nuclease and a deaminase enzyme that is specific for single-stranded DNA (ssDNA). The binding of the Cas9/gRNA to the target DNA strand leads to local denaturation of the DNA, called R-loop formation, essential for deamination. The deamination occurs in the non-targeted DNA strand in a restricted activity window of ~5 nucleotides in the ssDNA leading to a base change (see Figure 3). As detailed below, further modifications have been made to optimise the base editing window and increase the fidelity and efficiency of base editing.

### 4.1. Cytosine Base Editors

The first programmable base editor system to be developed for DNA was a fusion of dCas9 and the APOBEC1 cytidine deaminase which created a cytosine base editor (CBE) that could deaminate the amine of the target cytosine in positions 4–8 of the ssDNA on the non-target strand (see Figure 3), generating uracil [47]. This creates a U:G mismatch which is subsequently converted to a U:A base pair, or restored to a C:G by cellular DNA repair pathways. When DNA repair of the U:A base pair occurs, the uracil base is read as a thymine for both transcription and DNA replication. While the first version of the cytosine base editor (BE1) worked with an editing efficiency of 25–40% in vitro, Liu and colleagues observed much lower activity in human cells of 0.8–7.7%. The authors attributed this decrease to the cellular base excision repair (BER) pathway in which the uracil N-glycosylase (UNG) enzyme recognises the U:G mismatch intermediate and efficiently reverts it back to a C:G pair. To counter this, their second-generation cytidine base editor (BE2) also included an additional fusion domain comprising a uracil N-glycosylase inhibitor (UGI) to block BER. Replacement of dCas9 with Cas9 nickase (to create BE3) resulted in cutting of the non-edited strand of the DNA which favours the repair of the mismatch U:G to U:A (the desired edit) over the DNA repair back to C:G (the original unedited target).

An analogous cytosine base editing system was described by Nishida and co-workers [48]. Their system, called Target-AID, is similar to BE3 but instead of using APOBEC1 this group used cytidine deaminase 1 (CDA1) which acts in positions 2–4 of the ssDNA. Both groups [47,48] noted that, in some cases, C to G and C to A transversions are detected at the editing site, rather than the desired C to U transition, and these are most likely a consequence of BER, resulting in the insertion of a G or A residue. The addition of a second UGI domain to BE3 (to create BE4), was shown to reduce this [49].

Additional refinements to BE have been reported including modifications to (1) reduce indels by fusing the bacteriophage Mu-derived Gam protein (Mu-GAM) to BE4 to generate BE4-Gam [49]; (2) limit off-targets by the development of a high-fidelity version of BE3 [50]; (3) eliminate bystander products (edits of cytidines adjacent to the target residue) by introducing mutations in the APOBEC1 domain to narrow the base editing window (YE1-BE3, YE2-BE3, YEE-BE3 and eA3A-BE) [51] or, in contrast, increase their occurrence with a broader editing window (BE-PLUS) [52]; (4) the recognition of a wider range of targets by engineering variants of both SaCas9 and SpCas9, capable of recognising specific non-NGG PAMs (SaBE3, Sa(KKH)-BE3, VQR-BE3, VRER-BE3 and EQRBE3) [51]; or (5) a broad range of PAMs with an evolved variant of SpCas9-BE called BE-xCas9(3.7) [53]; (6) improve intracellular expression and nuclear localisation of the base editor (BE4max) [54]. Of note, Cas12a (Cpf1) variants of the C to U base editor with a broad PAM specificity have also been described [55].

### 4.2. Adenine Base Editors

A limitation of C to U base editing is that the majority of disease-causing SNPs originally occurred as a consequence of C to U deamination events, which often result in the formation of premature termination codons (PTCs—see Figure 2). Thus, there was a major incentive to develop a base editing strategy that could convert thymine to cytosine directly, or convert adenine to guanine on the opposite strand of DNA which would have the same effect as converting T to C (see Figure 3). In theory, the simplest approach would be the enzymatic deamination of adenine which would form inosine (I), a naturally occurring base which has a base pairing preference for guanine. The ability to correct A:T to G:C would have great therapeutic potential for genetic diseases. Indeed, it could correct nonsense mutations such as W1282X mutation, one of the most common CF-causing mutations and for which there are no specific drugs available.

The major obstacle to the development of the adenine base editors is that in Nature, there is no known adenine deaminase able to work in a DNA backbone. To address this challenge, Liu and co-workers [56] evolved one half of the dimeric *Escherichia coli* tRNA adenosine deaminase enzyme (TadA) that works in an RNA backbone in its bacterial host to function in a DNA context for genome editing. Then, to create a targetable adenine base editor (ABE), they fused a wild-type non-catalytic TadA monomer and an evolved TadA monomer with a Cas9 nickase. Using a number of target genes in human cell lines, they successfully demonstrated highly efficient base editing in a small window, converting any A:T to G:C in the positions ~4–7 (see Figure 3). Of several variants they created, ABE7.10 is the most efficient, whereas ABE7.9 and ABE6.3 can also edit in positions 8 or 9.

Adenine base editing shows a very high level of editing precision with A to C or A to T transversions observed at very low levels, most likely due to the much weaker ability of cells to remove inosine than uracil from DNA [57]. Moreover, the reduced incidence of inosine excision from edited strand prevents nicking of this strand which is thought to explain why A to I base editing shows much low indel frequencies compared to C to U base editing. Off-target studies in mutant mice suggest high specificity of ABE7.10, but there may be a low level of deamination from base editors that occurs in the absence of DNA-binding due to random encounters between deaminase domain of ABE and transient ssDNA intermediates [58].

## 5. DNA Base Editing—Proof-of-Concept Disease Models

Even though DNA base editing is a much more recently described technique than CRISPR/Cas9 gene editing, there are already several studies exploring its therapeutic potential for a diversity of diseases. Here, we describe three examples of in vivo base editing using either cytosine or adenine base editors to correct mutations that cause (5.1) Duchenne muscular dystrophy (Dmd); (5.2) phenylketonuria (PKU); or (5.3) hereditary tyrosinemia type I (HTI) in mouse models. We also describe two different studies that use cytosine base editing to inactivate the *Pcsk9* gene by introducing PTCs and show that this can permanently reduce blood cholesterol levels (5.4). These studies yield considerable insights into the in vivo use of base editing in terms of efficiency, indel frequency, off-target effects and delivery systems, all of which may be useful in developing the use of base editing for study and potential treatment of cystic fibrosis.

### 5.1. Adenine Base Editing of a Dmd Mouse Model

Duchenne muscular dystrophy is caused by mutations in the *Dmd* gene that encodes a protein called dystrophin expressed predominantly in skeletal and heart muscles cells. The loss of dystrophin leads to muscle atrophy and prevents Dmd patients performing the most basic tasks such as walking, standing or sitting, and as the disease progress, young boys and men affected by this disease die by respiratory or cardiac failure.

**Figure 4:**
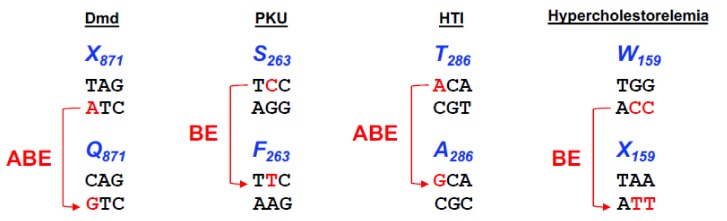
Base editing options to different diseases. Representation of the four different codons, X871, S263, T286, W159, responsible for causing Duchenne Muscular Dystrophy (Dmd), Phenylketonuria (PKU), Human Tyrosinemia type I (HTI) and Hypercholesterolemia, respectively. Nucleotide conversion is represented in blue and the base editor used in the studies to correct or introduce the mutation is represented in red.

To evaluate the therapeutic potential of base editing in vivo, Ryu and colleagues [59] delivered ABE7.10 using adeno-associated virus (AAV) to a mouse model of Dmd [60] that harbours a premature stop codon (Q871X) in the *Dmd* gene (see Figure 4). Given the limited packaging capacity of the adeno-associated virus of ~4.7 kb, the construct encoding ABE7.10 (its ORF alone is ~5 kb) plus its sgRNA had to be delivered in two parts using a dual trans-splicing adeno-associated virus (tsAAV) vector system. This system consists of two AAV vectors encoding either the N-terminal or C-terminal of ABE7.10 flanked by inverted terminal repeat (ITR) sequences. Following the transduction of cells by both vectors, the two mRNAs can trans-splice, resulting in expression of the full-length ABE7.10 protein and production of the guide RNA to form the base editing complex. Following the intramuscular administration of the vector system into the tibialis anterior muscle of the *Dmd* knockout mice, the level of precise A to G base substitutions were assessed by targeted deep-sequencing eight weeks post-injection. The PTC was converted to the glutamine codon with a frequency of 3.3 ± 0.9% and, more importantly, no unwanted indels or off-target events were detected at homologous sites which contained up to three mismatches. Remarkably, the *Dmd*-targeted tsAAV:ABE restored dystrophin expression in 17 ± 1% of myofibers as evaluated through histological analysis of the tibialis anterior muscle, a level which is sufficient to improve muscle function. The elevated level of functional correction relative to the frequency of editing may be due to a selective advantage for corrected myofiber cells in vivo.

### 5.2. Treatment of a Metabolic Disease by Cytosine Base Editing

Phenylketonuria is an autosomal recessive metabolic liver disease in which the impaired activity of the enzyme phenylalanine hydroxylase (PAH) leads to a decreased metabolism of phenylalanine (L-Phe) and its accumulation in blood, resulting in systemic hyperphenylalaninaemia. If PKU is not detected early, or not treated correctly with a phenylalanine-free diet, then patients, usually infants, will suffer from severe retardation, microcephaly, and seizures. Mice homozygous for mutations in the *Pah* gene fed on a normal diet which contains phenylalanine develop similar symptoms making them a suitable animal model to study PKU [61]. These Pah^enu2^ mice harbour a point mutation T:A to C:G (F263S) in exon 7 of the *Pah* gene that abolishes PAH enzyme function and causes abnormally elevated L-Phe levels (≥1500 µmol/L), compared to normal levels of ~120 µmol/l.

Villiger and colleagues [62] used a split AAV vector system to deliver the cytidine base editor nSaKKH-BE3 which includes a smaller Cas9 nickase from *Staphylococcus aureus* (Sa). The nSaKKH-BE3 base editor was split in two parts, p.N-int-BE3 containing the N-terminal half of the BE gene and p.C-int-BE3.sgRNA, which contained the C-terminal half of the BE gene, red fluorescent protein (RFP) sequence and the sgRNA specific for Pah^enu2^. In both cases, expression was driven by a synthetic liver-specific promoter P3 and constructs were packaged into pseudotyped AAV2/8 vectors (AAV8.N-int-BE3 and AAV8.C-int-BE3.sgRNA). Adult mice between eight and ten weeks of age received tail-vein injections of either low (5 × 10^10^ vector genomes (vg)) or high dose (5 × 10^11^ vg) per mouse. As a control, the AAV8.C-int-BE3.sgRNA was replaced by a modified vector lacking the Pah^enu2^-specific sgRNA (AAV8.C-int-BE3). The specificity of the P3 promoter was confirmed by the detection of RFP expression only in liver and not in other organs. In the low-dose group, blood L-Phe levels were moderately reduced to ~1000 µmol/L at eight weeks post-injection, while, in the high-dose group, L-Phe levels were reduced to physiological normal levels of ≥120 µmol/L after only six weeks post-injection and remained at this level for up to 26 weeks.

Quantification and precision of *Pah* gene correction rates were assessed by the high throughput sequencing (HTS) of PCR amplicons from genomic DNA [62]. The editing levels in mice measured at 4, 8, 14, and 26 weeks post-injection showed 10, 19, 22 and 25% C-to-T correction rates, respectively, whereas indels rose to a maximum of 10% at 26 weeks post-injection. Of note, editing at the DNA level gave rise to a higher level of edited transcripts, with levels of 17, 34, 39 and 44% at corresponding time time-points. Analysis of whole liver lysates showed the restoration of wild-type enzyme activity that correlated to correction rates of mRNA and genomic DNA. Furthermore, PKU-associated phenotypes such as reduced weight and hypopigmentation were reversed in corrected mice. Ten computationally predicted off-target loci, were analysed in mice eight weeks after the administration of a higher dose of AAV and no C to T conversion or indels above background were detected by HTS. In addition, no indication of excessive DNA damage or cell proliferation after prolonged exposure to low levels of base editors was observed [62]. Together, this approach provides proof-of-principle that the AAV-mediated delivery of base editing machinery allows the rescue of the inborn metabolic disease of liver, an adult tissue with limited proliferative capacity.

### 5.3. Adenine Base Editing of a Mouse Model of Hereditary Tyrosinemia I

Hereditary tyrosinemia type I is a fatal genetic disease caused by loss-of-function of fumarylacetoacetate hydrolase (FAH) enzyme, a key protein in the tyrosine metabolic pathway. When FAH enzymatic activity is compromised, this leads to the accumulation of toxic metabolic intermediates which causes hepatocyte apoptosis and, consequently, severe liver damage. The existing Fah^mut/mut^ mouse model [63] carries a homozygous G-to-A point mutation (A236T) in the last nucleotide of exon 8 (see Figure 4), resulting in exon skipping and loss of FAH protein. When these mice are supplemented with 2-(2-nitro-4-trifluoromethylbenzoyl)-1,3-cyclohexanedione (NTBC), an inhibitor of the tyrosine metabolism upstream of FAH, toxin build-up and liver damage is prevented in these animals.

In an early study using conventional Cas9 gene editing by HDR, it was established that corrected liver cells have a selective advantage and expand to repopulate the liver [64]. A previously validated Fah sgRNA from that study, where the disease-causing mutation is located at position 9, and base editor ABE6.3, which performs better at this position, were used to edit the *Fah* gene [65]. As noted by the authors, there is other “A” in the sgRNA position 6 whose editing changes a serine codon into alanine. This change might not restore splicing but its proximity to the Fah enzyme active site may compromise enzyme activity and impede the functional rescue of edited hepatocytes. Both ABE6.3 and Fah sgRNA plasmids were delivered to adult mice liver through hydrodynamic tail vein injection at day 1. When NTBC supplemented-water was removed at day 6 to promote HTI, there was a rapid loss of 20% of total body weight in control mice (injected with PBS or ABE6.3 alone), while the injection of both ABE6.3 and Fah sgRNA rescued weight loss and remained until 106 days after injection without NTBC treatment. Expansion of corrected hepatocytes, as shown by immunohistochemistry (IHC) of liver sections with FAH-specific antibody, contributed to the weight loss rescue. This data demonstrates not only the viability and functionality of ABE corrected hepatocytes at a long-term, but also indicates that ABE is able to rescue the disease phenotype in vivo.

To understand whether ABE corrects *Fah* splicing mutation in exon 8 in ABE-treated mice (post-NTBC withdrawal and hepatocyte expansion), the authors deep-sequenced a genomic region spanning exon 5 to 9. This data showed a correction rate of 9.5 ± 4.0% and of 1.9 ± 0.9% at either position 9 or position 6, respectively. The correction rate at both positions was ~0.1%. The rate of stochastic insertions and deletions was as low as 0.05% in ABE-treated hepatocytes. Deep sequencing of the four top-ranking off-target sites showed no A:T to G:C editing in ABE-treated livers. However, the authors consider that on-target ABE efficiency in vivo is relatively low making the detection of off-target effects difficult.

To improve editing efficiency, ABE6.3 was used with a codon optimized Cas9 nickase and a N-terminal nuclear localization sequence (NLS), hereinafter called the RA6.3 base editor. The injection of RA6.3 and Fah sgRNA in the same animal model showed more FAH^+^ hepatocytes and higher A to G correction rate than when ABE6.3 was used, as confirmed by IHC and deep sequencing. The authors then tested the delivery of RA6.3 mRNA along with a chemically modified sgRNA to the mice liver trough the tail vein injection of lipid nanoparticle. To measure the initial ABE efficiency, mice were kept in NTBC treatment to prevent hepatocyte proliferation. The IHC results showed no edited hepatocytes in control Fah^mut/mut^ mice, while mice injected with both RA6.3 mRNA and Fah sgRNA showed 0.44% ± 0.28% of edited hepatocytes. This data suggests that the non-viral delivery of ABE is possible in the adult mouse liver. However, improvements in ABE mRNA stability and delivery vehicles are needed.

Once again, this study shows the potential of ABE to correct A to G point mutations in adult animal models rescuing the disease phenotype, with a very low rate of indel formation. This study also demonstrates how improvements in base editors, in combination with better delivery systems, can increase editing efficiency in vivo.

### 5.4. Cytosine Base Editing of PCSK9 as a Permanent Therapeutic Approach

High levels of low-density lipoprotein (LDL) cholesterol in circulation is a well-established risk factor for cardiovascular disease, the leading cause of death worldwide. Currently, the main treatment for hypercholesterolemia is statins but some patients are intolerant to this drug. An alternative approach to reduce cholesterol levels is the inhibition of proprotein convertase subtilisin/kexin type 9 (PCSK9). This protein has a key role in cholesterol homeostasis by directing membrane-bound LDL receptors to lysosomal degradation. Individuals with naturally occurring loss-of-function mutation in *PCSK9* gene have lower levels of LDL cholesterol compared to general population. Human *PCSK9*-targeted therapies, such as monoclonal antibodies and siRNAs, have been used in clinical trials but their benefit is short and chronic administration is required. Recent studies using CRISPR-Cas9 NHEJ to disrupt *Pcsk9* gene in mouse and human hepatocytes in vivo had demonstrated reduced plasma concentrations of PCSK9 protein and cholesterol [66,67].

The first study uses cytosine base editor BE3 to disrupt the *Pcsk9* gene in adult mice by introducing a nonsense mutation (W159X) [68]. A single adenoviral vector was used to deliver BE3 and the sgRNA targeting W159 codon in the *Pcsk9* gene (BE3-Pcsk9) and administered through retro-orbital injection to 5-week-old male C57BL/6J mice. The base editing of the *Pcsk9* gene resulted in a reduction of 56 and 28% in the PCSK9 protein and cholesterol plasma levels, respectively, and it greatly increased hepatic LDL receptor levels. Deep sequencing of the genomic DNA samples from mice five days after treatment, showed 34% base editing of alleles in the liver and an indel rate of ~1%. These editing and indel levels were stable for at least four weeks after treatment. It is important to note that other changes rather than C to T were observed, resulting in missense variants instead of nonsense variants in some alleles; but, on average, 22% of alleles were precisely edited to W159X. Nine potential base editing off-target sites were evaluated by deep sequencing in three BE3-Pcsk9-treated and one control mouse, but no evidence of off-target C-to-T edits, nor indels, were observed.

In a more recent study, researchers generated a knock-in (KI) mouse with liver-specific expression of human PCSK9 driven by albumin promoter, termed hPCSK9-KI, to model of human hypercholesterolemia [69]. Results show a hypercholesterolemic phenotype driven by PCSK9 that gets worse with age, similar to what has been seen in human hypercholesterolemia. Significantly lower plasma cholesterol levels in hPCSK9-KI but not in control mice, after the administration of evolocumab (an inhibitor of human PCSK9), support the hypothesis that the hypercholesterolemic phenotype observed is dictated by the liver-specific expression of human PCSK9. To demonstrate the efficiency of human *PCSK9* gene base editing in this in vivo system, BE3 base editor and a previously validated sgRNA (therefore termed gMH) were used [68]. This sgRNA has perfect complementarity to the mouse *Pcsk9* locus and one nucleotide mismatch to the human gene. The hPCSK9-KI 10-week-old mice were injected via tail vein with adenoviral vectors encoding BE3 together with gMH or BE3 alone as control. Three weeks after injection, it was observed in treated mice that most of the single nucleotide substitutions were the targeted C to T transitions in both human *PCSK9* and mouse *Pcsk9* by deep sequencing of the genomic DNA from liver tissue. Significant reductions were also observed at three weeks post-injection in (1) circulating levels of both human and mouse PCSK9 protein and (2) total cholesterol plasma levels. In addition, an increase in LDL receptor-positive cells was detected by IHC in BE3-gMH injected mice.

Importantly, this study compares the use of the same sgRNA with both CRISPR/Cas9 and base editing techniques. It shows that base editing can be used in vivo to introduce precise point mutations without any detectable off-targets and a significantly lower frequency of indels when compared to Cas9-mediated genome editing.

### 5.5. Base Editing as a Therapeutic Option for CF

These studies demonstrate the capacity of both the DNA-specific CBE and ABE proteins to correct or introduce C to T or A to G point mutations, respectively, which could be applied to a large number of CF-causing variants. Indeed, base editing of *CFTR* variants is not a new approach per se, as Rosenthal and colleagues reported site-specific RNA base editing of the *CFTR* W496X mRNA which transiently restored CFTR function in Xenopus oocytes [70], but this may have limited therapeutic application as it would require long-term expression of the editor and gRNA.

For the correction of CF-causing mutations in genomic DNA, the base editing approach could be particularly useful for two major categories of mutation, PTCs and certain splicing mutations. Adenine base editing will convert a PTC to one of four possible codons (TGG = Tryptophan, CGA/CGG = Arginine, or Glutamine) depending on the sequence of the PTC and which strand is targeted. Thus, W1282X could be repaired to the wildtype sequence and should therefore restore full function, whereas G542X would be repaired to a Tryptophan or Arginine codon which should stop nonsense-mediated decay of the variant mRNA, and make a full-length CFTR protein, but it may show reduced function due to change in amino acid sequence. It should be noted that the target A residues for both these mutations lie outside the editing window for currently available ABE proteins.

For the correction of splicing mutations, ABE could in principle repair mutations such as 1717-1G>A by targeting the coding strand, whereas CBE could repair mutations such as 3272-26A->G by targeting the non-coding strand (conversion of C to T on non-coding strand would lead to conversion of G to A on the coding strand).

As discussed in detail above, the base editing approach is compatible with a wide range of methods including plasmids, RNA molecules, RNP–lipid complexes [71]. It can also be delivered by AAV, with the base editor cDNA split across two vectors to generate a trans-spliced mRNA. An advantage relative to gene editing by HDR is that no donor is required, but the downside is that not all changes can be corrected. Also, the target A or C must be in the correct editing window relative to the PAM site, but with an increasing range of base editors with altered PAM specificities, this is becoming less of a challenge. Off-target effects of base editors are less well studied, but in principle the much lower rate of indels should reduce potential genomic rearrangements, and early studies suggest that ABE off-targets are lower than CBE in genome DNA [72,73,74]. Of note, a recent study has detected the deamination of both cytosines and adenines in the RNA of human cells when treated with DNA base editors [75]. Even though these base editing-induced RNA edits can occur in both protein-coding and non-protein coding sequences they are short-lived. The incidence of this RNA editing can be reduced when base editing is performed with RNPs rather than plasmid encoded base editors. Moreover, Grünewald and colleagues developed two BE variants that further reduced the likelihood of RNA editing events.

In summary, there are a broad array of gene-editing techniques available to correct many CF-causing variants simultaneously using superexon donors or targeted integration of the complete CFTR cDNA, or both gene and base editing techniques that could be used to precisely repair individual CF-causing variants. As we approach a situation where small-molecule therapies are potentially available for more than 90% of CF patients, there is a growing urgency for the development of therapeutic strategies for people with rarer complex *CFTR* variants. If it is possible to establish proof-of-concept in one or more the family of CF animal models for this approach, and determine the most efficient and safe method of in vivo or ex vivo delivery, then this would pave the way for the evaluation of a personalised gene or base editing strategy as a potential long-term therapeutic strategy for this disease.
